# Modeling of the Coral Microbiome: the Influence of Temperature and Microbial Network

**DOI:** 10.1128/mBio.02691-19

**Published:** 2020-03-03

**Authors:** Laís F. O. Lima, Maya Weissman, Micheal Reed, Bhavya Papudeshi, Amanda T. Alker, Megan M. Morris, Robert A. Edwards, Samantha J. de Putron, Naveen K. Vaidya, Elizabeth A. Dinsdale

**Affiliations:** a Department of Biology, San Diego State University, San Diego, California, USA; b College of Biological Sciences, University of California Davis, Davis, California, USA; c Department of Mathematics and Statistics, San Diego State University, San Diego, California, USA; d National Center for Genome Analysis Support, Pervasive Institute of Technology, Indiana University, Bloomington, Indiana, USA; e Viral Information Institute, San Diego State University, San Diego, California, USA; f Bermuda Institute of Ocean Sciences, St. George’s, Bermuda; Pennsylvania State University; University of Hawaii at Manoa

**Keywords:** host-microbe, metagenomics, microbial communities

## Abstract

Host-associated microbial communities are shaped by extrinsic and intrinsic factors to the holobiont organism. Environmental factors and microbe-microbe interactions act simultaneously on the microbial community structure, making the microbiome dynamics challenging to predict. The coral microbiome is essential to the health of coral reefs and sensitive to environmental changes. Here, we develop a dynamic model to determine the microbial community structure associated with the surface mucus layer (SML) of corals using temperature as an extrinsic factor and microbial network as an intrinsic factor. The model was validated by comparing the predicted relative abundances of microbial taxa to the relative abundances of microbial taxa from the sample data. The SML microbiome from Pseudodiploria strigosa was collected across reef zones in Bermuda, where inner and outer reefs are exposed to distinct thermal profiles. A shotgun metagenomics approach was used to describe the taxonomic composition and the microbial network of the coral SML microbiome. By simulating the annual temperature fluctuations at each reef zone, the model output is statistically identical to the observed data. The model was further applied to six scenarios that combined different profiles of temperature and microbial network to investigate the influence of each of these two factors on the model accuracy. The SML microbiome was best predicted by model scenarios with the temperature profile that was closest to the local thermal environment, regardless of the microbial network profile. Our model shows that the SML microbiome of *P. strigosa* in Bermuda is primarily structured by seasonal fluctuations in temperature at a reef scale, while the microbial network is a secondary driver.

## Abstract (precis)

Coral microbiome dysbiosis (i.e., shifts in the microbial community structure or complete loss of microbial symbionts) caused by environmental changes is a key player in the decline of coral health worldwide. Multiple factors in the water column and the surrounding biological community influence the dynamics of the coral microbiome. However, by including only temperature as an external factor, our model proved to be successful in describing the microbial community associated with the surface mucus layer (SML) of the coral *P. strigosa*. The dynamic model developed and validated in this study is a potential tool to predict the coral microbiome under different temperature conditions.

## INTRODUCTION

The community structure of a host-associated microbiome is shaped by factors that are both extrinsic (e.g., abiotic conditions and community composition of micro- and macroorganisms in the surrounding environment) and intrinsic (e.g., microbial interactions and host physiology) to the holobiont (1–4). Identifying the role that each factor plays in predicting the diversity and community structure in the microbiome of host organisms is a major priority in microbial ecology, especially in the context of environmental changes (5–8).

Coral reefs are among the most productive, biodiverse, and endangered ecosystems in the world (9, 10), and the health of the reefs is directly mediated by the associated microbiota (11–14). Corals host one of the most phylogenetically diverse microbiomes among animal hosts (15), which is composed by endosymbiotic dinoflagellates (*Symbiodiniaceae*), bacteria, archaea, fungi, and viruses (16). The coral microbiome provides essential services to the holobiont, such as nutrient cycling (17–20) and protection against opportunistic pathogens via competition and the production of antibiotic compounds (21–23).

The symbiotic relationships in the coral holobiont are sensitive to changes in environmental conditions. Extrinsic factors, including eutrophication (24, 25), salinity (26), pH (27, 28), neighboring macroorganisms (4), herbivorous fish abundance (29), copper concentration (30), and temperature (27, 31–33), alter the taxonomic and functional composition of the coral microbiome. Overall, the response of the coral microbiome to environmental disturbances is consistent across multiple stressors, characterized by an increase in the relative abundances of *Vibrionales*, *Flavobacteriales*, *Rhodobacterales*, and *Alteromonadales* (33).

Corals are widely recognized as thermally sensitive organisms (34–37), and elevated temperatures are correlated with coral bleaching (i.e., loss of the algal symbiont) and disease outbreaks in coral reefs worldwide (32, 38–42). High seawater temperature causes the greatest change in the functional metabolism of coral microbiome, compared to eutrophication and low pH, as a result of an increase in the abundance of *Vibrio* spp. and other diseased-associated microbes (27). Seawater temperature, therefore, is one of the most important drivers of the coral-microbial community composition (43).

The surface mucus layer (SML) microbiome constitutes the direct interface between the coral host and the environment. Within the coral holobiont, the coral SML, tissue, and skeleton provide different microhabitats to the microbial community (44, 45). Across the three coral microhabitats, the microbial composition of the SML is the compartment that is most influenced by environmental factors (e.g., temperature, benthic coverage, and geographic region) (44) and by the microbial community in the water column (e.g., high similarity) (45).

The influence of factors that are intrinsic to the coral holobiont on regulating the microbiome is less clear. Host genotype and *Symbiodiniaceae* phylotype are among intrinsic factors that do not correlate with the taxonomic composition in the coral microbiome, but instead the microbiome correlates with environmental factors such as habitat and seasonality (46–48). Microbe-microbe interactions (e.g., competition, predation, mutualism), however, are intrinsic factors that are potentially major drivers of the coral microbial community structure and holobiont homeostasis (20, 49–52). Coral-associated bacteria produce inhibitory compounds and have antagonist effects on each other, including *Pseudoalteromonas* spp. inhibiting the coral pathogen Vibrio shiloi (51). High temperatures, however, can change the way microbes interact (53, 54). The number of coral-bacterial isolates inhibited by Alphaproteobacteria is drastically reduced when temperature increases from 25 to 31°C (51). Therefore, temperature and microbial interactions are interconnected and act simultaneously in shaping the community structure of the coral microbiome.

Mathematical models that use microbial growth rates as a function of environmental temperature (55–59) and include microbial interactions derived from network analysis (60, 61) can be a powerful tool to investigate the dynamics of microbial communities. However, this approach remains to be further adapted and applied to coral reef systems. The ecological interactions between the members of the microbiome are challenging to elucidate, but metagenomic sequencing (62–64), combined with network analysis, has been able to reveal these relationships (65–69). Microbial networks constructed by correlation-based methods identify microbial interactions and the key taxa to the structure of the community by using measures of network centrality, such as eigenvector and betweenness centrality (70, 71).

Here, we develop a new differential equation mathematical model to determine the community structure of the microbiome associated with coral SML using temperature as an extrinsic factor and microbial network as an intrinsic factor to the coral holobiont. To provide the input data for the model development and validation, we selected the coral reefs of Bermuda, where coral colonies are exposed to different thermal regimes at a reef scale. The reef system in Bermuda is formed by distinct physiographic reef zones, and there is a pronounced spatial gradient in temperature profiles across the inner and outer reef zones. The seawater temperature differences of the shallow inner lagoon reefs range between 13 and 15°C (winter averages of 16 to 17°C and summer averages of 30 to 31°C), whereas the outer reef temperature range is moderated with a 10°C temperature difference (seasonal averages of 19 and 29°C, respectively) (72). The temperature profiles specific to each reef zone were simulated in the model. Metagenomic analysis was used to describe the taxonomic composition and generate the microbial network of the SML microbiome associated with the coral Pseudodiploria strigosa (Dana, 1846) from inner and outer reefs. The model was validated by comparing the predicted relative abundances of each microbial class to the measured relative abundances of each microbial class. Finally, the model was applied to six scenarios that combine different profiles of temperature and microbial network to investigate the drivers of the coral-microbial community dynamics. Our study shows that the SML microbiome of *P. strigosa* in Bermuda is primarily structured by reef-scale seasonal fluctuations in temperature, while the microbial network is a secondary driver.

## RESULTS

### Microbial community in the coral SML.

**FIG 1:**
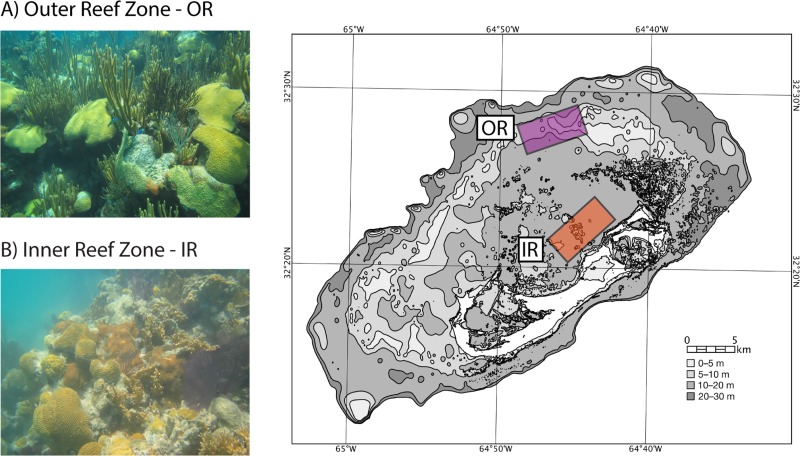
The coral reef in the Bermuda archipelago is composed of different reef zones across the platform. The outer rim reef (OR) is a relatively more stable thermal environment compared to the inner lagoon patch reefs (IR). Each reef zone was replicated (*n* = 6 corals per zone) in the colored areas.

**FIG 2:**
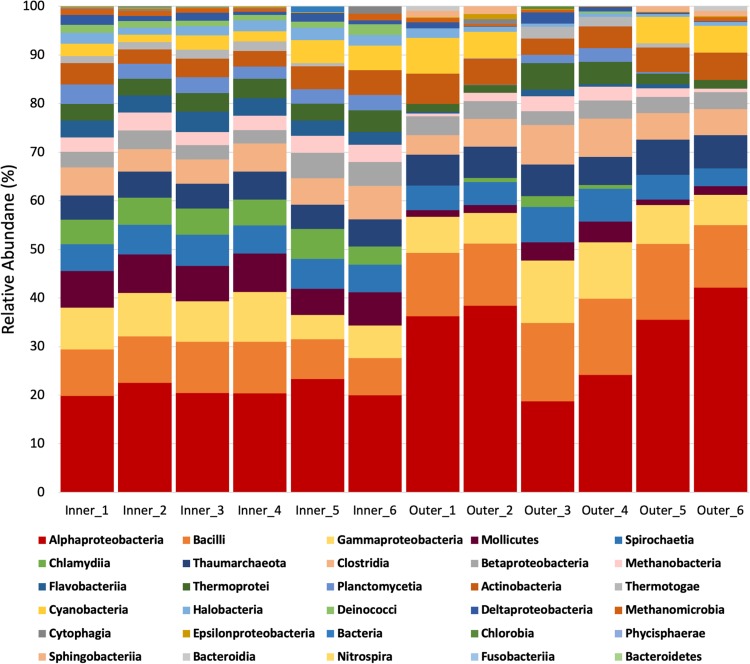
Relative abundances of microbial classes associated with coral SML from inner and outer reefs.

**FIG 3:**
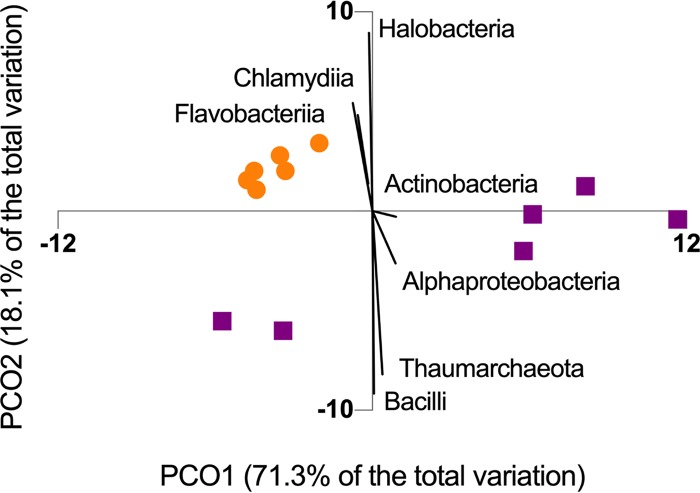
The SML microbiome of *P. strigosa* corals from the inner reefs (circles) showed greater clustering than corals from the outer reef (squares), visualized using a principal coordinate analysis of relative abundance of microbial classes. Vectors correspond to Spearman’s correlation indices higher than 0.9.

The structure of the SML microbiome of *P. strigosa* was specific to each reef zone (Fig. 1) in terms of the relative abundances and microbial network parameters. The SML microbiome of *P. strigosa* included 30 bacterial and archaeal classes (inner reefs = 23, outer reefs = 21), with high proportional abundances of *Alphaproteobacteria*, *Bacilli*, and *Gammaproteobacteria* (Fig. 2). Inner and outer reef microbial communities shared the same codominant classes, but the relative abundances of taxa were significantly different between reef types (PERMANOVA, Pseudo-F = 7.79; P(perm) = 0.004; see Table S1 in the supplemental material). The sample metagenomes from inner and outer reefs formed separate clusters, indicating that *P. strigosa* harbors a reef zone-specific SML microbiome (Fig. 3). There was lower intercolony variability in the SML microbial community within the inner reef corals compared to the outer reef corals (SIMPER, average similarity, inner = 92.9%, outer = 84.7%, see Table S2a and b). Therefore, the coral SML microbiome structure is more homogenous across host individuals of the same species in a more fluctuating environment than in a more stable environment in Bermuda. The inner and outer coral-mucus microbiome had an average dissimilarity of 18.16% (Table S2c). The main classes contributing to the dissimilarity between reef zones were *Chlamydiia*, *Deinococci*, and *Flavobacteriia*, which were overrepresented in the microbiome of corals from inner reefs.

10.1128/mBio.02691-19.3 TABLE S1 PERMANOVA table of results using a Bray-Curtis similarity matrix of fourth-root transformed relative abundances from metagenomes collected from outer and inner reefs. The permutation method selected was unrestricted permutation of raw data using 999 permutations. The reef zone was treated as a fixed factor of two levels. Download Table S1, DOCX file, 0.01 MB. Copyright © 2020 Lima et al. 2020 Lima et al. This content is distributed under the terms of the Creative Commons Attribution 4.0 International license.

10.1128/mBio.02691-19.4 TABLE S2 Similarity percentages (SIMPER) of species contributions using a Bray-Curtis similarity matrix of fourth-root transformed relative abundances from metagenomes collected from outer and inner reefs. Each reef zone was treated as a factor group. Cutoff for low contributions, 90.00%. The SIMPER results of similarity within groups is shown for outer reef (a), inner reefs (b), and a comparison between the two reef zones (c). (a) Group outer, average similarity: 84.70; (b) group inner, average similarity: 92.89; (c) groups outer and inner, average dissimilarity = 18.16. Download Table S2, DOCX file, 0.02 MB. Copyright © 2020 Lima et al. 2020 Lima et al. This content is distributed under the terms of the Creative Commons Attribution 4.0 International license.

**FIG 4:**
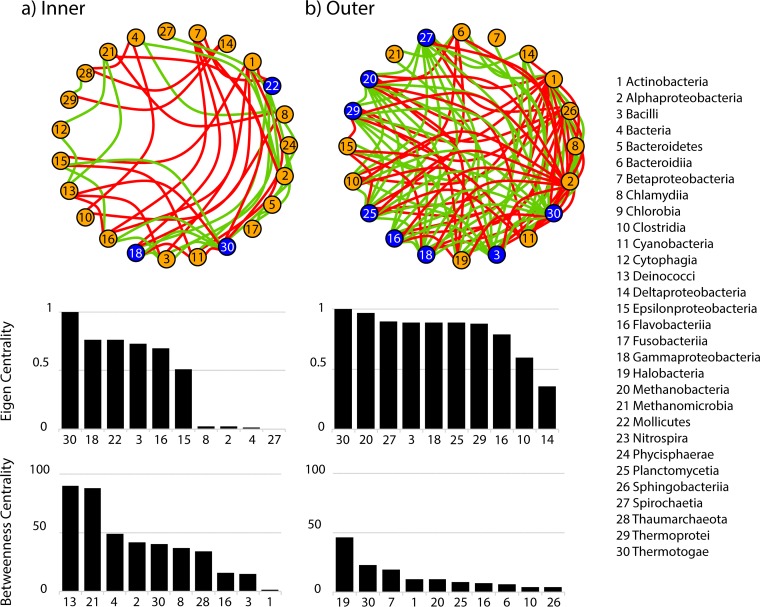
Network analysis of the coral SML microbiome of *P. strigosa* from inner (A) and outer (B) reefs. Each node represents a microbial class interconnected by positive correlations (green) and negative correlations (red) (Spearman’s rho > 0.7). Nodes that have an eigen centrality higher than 0.75 are highlighted in blue. The top 10 values of eigen centrality and betweenness centrality across microbial classes are graphed below each network.

The SML microbiome from each reef zone showed a specific network (Fig. 4). The microbial network from inner reefs had 23 nodes (i.e., microbial classes), 46 edges, and a diameter of 7, while the microbial network from outer reefs had 20 nodes, 94 edges, and a diameter of 3. The network in the outer coral SML microbiome was more tightly connected compared to the inner coral SML microbiome taxa (eigen centrality > 0.75, *n* = 8 classes in outer reefs and *n* = 3 classes in inner reefs; Fig. 4). High values of eigen centrality characterize a highly structured community network, in which the relative abundances of microbial taxa are tightly correlated (73). On the other hand, the microbial network from inner reefs showed higher betweenness centrality and lower eigen centrality (maximum betweenness = 45 in outer reefs and 90 in inner reefs; Fig. 4). A microbial class with a high level of betweenness centrality sits at a position that is important in facilitating the connectivity of the network (74). *Thermotogae*, even though it was a rare class (average relative abundance of <1%) showed high eigen and betweenness in both reefs. *Methanobacteria* is a key node to the network of coral microbiome in outer reefs, with both high eigen centrality and betweenness centrality (Fig. 4). In the microbiome of corals from inner reefs, *Gammaproteobacteria*, *Mollicutes*, *Bacilli*, and *Flavobacteriia* showed the highest eigen centrality, and *Deinococci*, *Methanomicrobia*, and *Alphaproteobacteria* showed the highest betweenness centrality in the community network (Fig. 4).

### Modeling the coral microbiome.

We developed a dynamic model based on differential equations to describe the relative abundances in the microbial community associated with the coral mucus in response to temperature and microbial network. The model uses classical logistic growth equations to calculate growth rates for each microbial class as a function of seawater temperature over time (59) and accounts for the effects of microbial interactions on growth rates using network correlation coefficients (60). The model was validated by comparing model prediction with sample data, using the classes identified in the metagenomes that showed an average relative abundance greater than 1% in at least one of the two reef zones (*n* = 17 classes shared between reef zones, out of the total of 23 in the inner reefs and 21 in the outer reefs). For both the inner and outer reefs, we used the corresponding specific networks and corresponding temperature profiles into the model of the SML microbiome of *P. strigosa* and solved the model to predict the microbiome composition (Fig. S1). Linear regression of the sample data and the model prediction of abundance of each microbial class had a slope of 0.96 and an intercept of 0.45, which were not statistically significant different from one and zero, respectively (Wald linear hypothesis test, sum of squares = 8.52, F = 0.81, *P* = 0.55). Therefore, the mathematical model developed in this study was accurate in predicting the observed SML microbiome of *P. strigosa* from the sample data, and the approach implemented here is appropriate for modeling the coral microbiome.

10.1128/mBio.02691-19.1 FIG S1 Model prediction versus experimental data of relative abundance (%) of bacteria classes (most abundant identified from mucus metagenomes) in inner (*n* = 17 classes) and outer (*n* = 17 classes) reef zones. Download FIG S1, TIF file, 2.2 MB. Copyright © 2020 Lima et al. 2020 Lima et al. This content is distributed under the terms of the Creative Commons Attribution 4.0 International license.

### Investigating the role of temperature and microbial network as model components for the coral microbiome.

**FIG 5:**
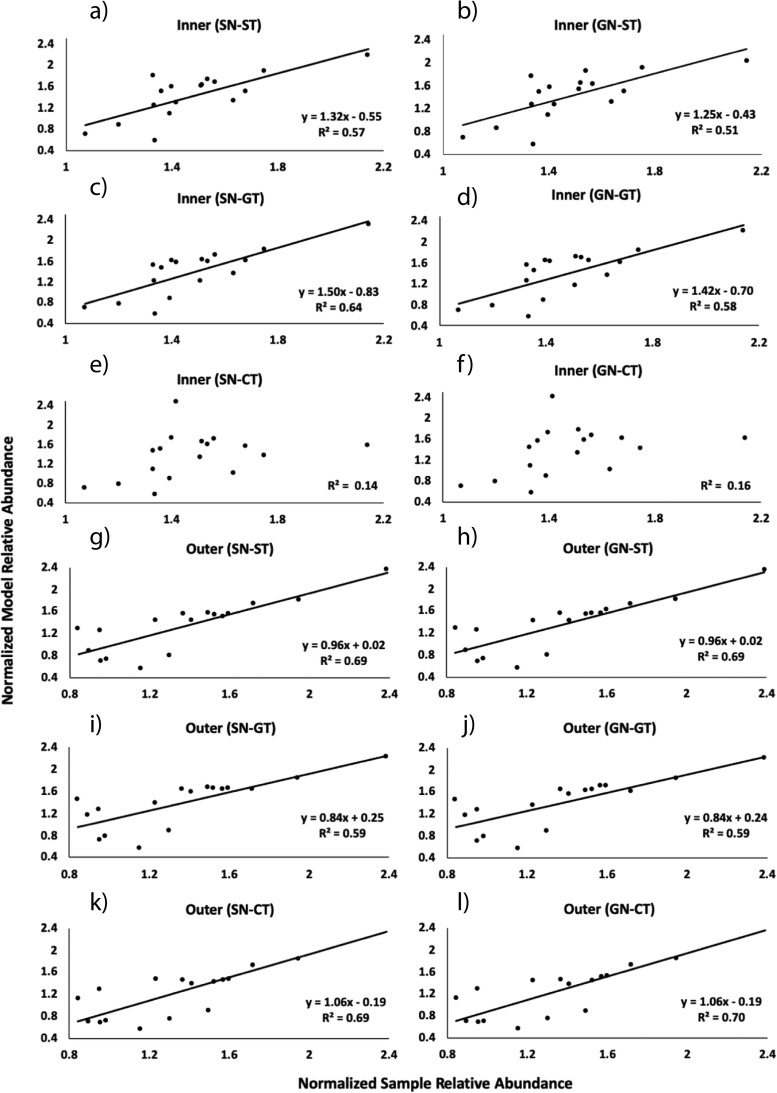
Linear regression analysis between sample and model data based on fourth-root transformed relative abundances. The sample data corresponds to the most abundant microbial classes (*n* = 17, average abundance >1%) in the metagenomes sequenced from surface mucus layer of the coral *P. strigosa* (*n* = 12 colonies; 6 per reef zone). The model abundances of these same classes were generated by the mathematical model for both inner (a to f) and outer reefs (g to l) using six different scenarios. The solid lines represent significant linear regressions (ANOVA, *P* < 0.05).

We used our model to determine the key drivers governing the community structure in the coral SML microbiome. Six different combinations of temperature profiles (T = fluctuating temperature; CT = constant temperature) and network structures (N) that were both either specific to the reef zone (S) or generalized to the coral reef system (G) were evaluated (Table 1). An example scenario is SN-ST, which combines specific network and specific fluctuating temperature. The same model scenarios were applied to the microbiome associated with each reef zone (inner and outer reefs) separately generating twelve corresponding model outputs total (Fig. 5). Ten of the total of twelve model outputs analyzed had a significant linear regression between sample data and model predictions (Fig. 5a to d and g to l). Model outputs that significantly described the relative abundances of sample data produced *R*^2^ values ranging from 0.51 to 0.70 (Table S3). Model scenarios that used constant temperature profiles were not significantly correlated with the coral microbiome from inner reefs (Fig. 5e to f, inner SN-CT, GN-CT, *R*^2^ = 0.1). Therefore, the coral SML microbiome from inner reefs could not be successfully predicted by our model under constant temperatures, regardless of the network used. In contrast, when the temperature was kept constant in outer reefs, the model output accurately described the measured microbiome.

**TABLE 1:**
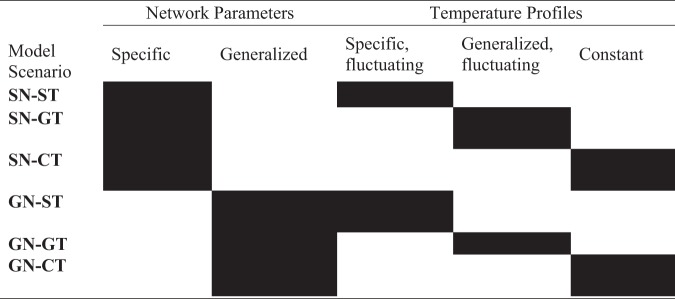
Model scenarios generated by different combinations of network parameters and temperature profiles

10.1128/mBio.02691-19.5 TABLE S3 Results of the linear regression analysis between the model outputs and observed data after fourth-root transformation to achieve normality. Download Table S3, DOCX file, 0.01 MB. Copyright © 2020 Lima et al. 2020 Lima et al. This content is distributed under the terms of the Creative Commons Attribution 4.0 International license.

Among the ten model scenarios that fit the linear regression analysis, some combinations of microbial network and seawater temperature profiles generated more accurate outputs (higher *R*^2^) than others. In inner reefs, the greatest accuracy is achieved by using a specific network and an average fluctuating temperature profile (Fig. 5c, inner SN-GT, *R*^2^ = 0.64). The accuracy of the model output is lower when a generalized network and a warmer and more fluctuating temperature profile is applied (Fig. 5b, inner GN-ST, *R*^2^ = 0.51). The microbiome of corals from the outer reefs of Bermuda was best predicted by the model compared to inner reefs since all model scenarios produced significant linear regressions. The outputs produced by the model scenarios that used milder fluctuating temperature profiles (Fig. 5g and h, outer SN-ST, GN-ST) and constant temperature profiles (Fig. 5k and l, outer SN-CT, GN-CT) showed similar accuracies (*R*^2^ = 0.69), regardless of the network profile. Model scenarios that used an average fluctuating temperature profile (Fig. 5i and j, outer SN-GT, GN-GT) generated outputs with lower accuracy (*R*^2^ = 0.59) in outer reefs.

**FIG 6:**
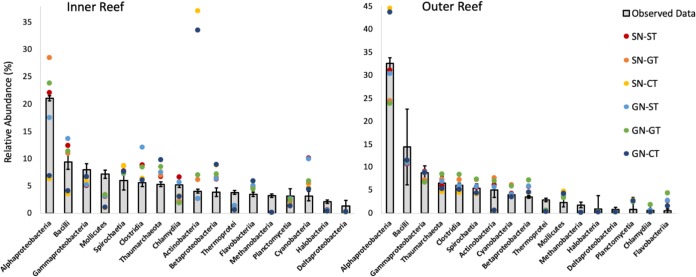
Model predictions of the relative abundances of seventeen microbial classes generated using the six scenarios (SN-ST, SN-GT, SN-CT, GN-ST, GN-GT, and GN-CT) compared to the observed data (means ± the standard deviations; *n* = 6 per reef zone) for the inner and outer reef zones, respectively.

The accuracy of the model relative abundances in the coral SML microbiome varied across taxa between the two reef zones (Fig. 6). *Alphaproteobacteria*, the most proportionally abundant taxon in the coral SML microbiomes of both reef zones, was more accurately predicted by model scenarios that used reef-specific temperature profiles (Fig. 6, SN-ST, GN-ST). On the other hand, model scenarios that used constant temperature profiles (Fig. 6, Inner SN-CT, GN-CT) were the least accurate when modeling the abundance of *Alphaproteobacteria*, causing underestimation in inner reefs and overestimation in outer reefs. Other codominant taxa, such as *Bacilli* and *Mollicutes*, were also underrepresented in the “CT” model scenarios in inner reefs. *Actinobacteria* was overestimated by approximately 8-fold under constant temperatures in inner reefs (Fig. 6, inner SN-CT, GN-CT). The model scenarios applied to the coral SML microbiome of outer reefs produced outputs that were within the range of the standard deviation from the mean relative abundances of the observed data for most of the microbial classes (Fig. 6).

## DISCUSSION

**FIG 7:**
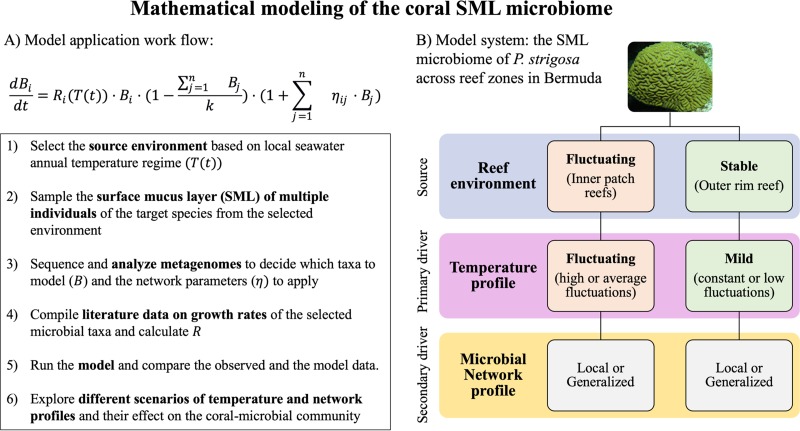
Modeling the coral surface mucus layer (SML) microbiome. (Left) Suggested workflow to apply the model developed in this study. (Right) Conceptual schematic of the drivers of the microbial community structure within the mucus of *P. strigosa* from each reef environment in Bermuda. The seawater temperature profile is the primary driver predicting the coral microbiome structure associated with different reef zones. Greater accuracy between the model and sample data were achieved when the model temperature profile depicts the natural temperature regimes. The network profile, used as a proxy for the microbial community interactions, is considered a secondary driver since it did not influence the accuracy of the model scenarios.

The mathematical model we developed predicted the microbial relative abundances in the SML microbiome of the coral *P. strigosa* and is a robust tool for investigating the effects of different profiles of temperature and microbial network on the model microbiome. We considered temperature as the major driver affecting the predictability of the coral SML microbiome of both reef zones compared to microbial network (Fig. 7). Differences in accuracies were driven by the distinct temperature profiles used across the model scenarios, while different microbial network profiles caused no apparent effect. The SML microbiome was best predicted by model scenarios that had a temperature profile that is closest to the local thermal environment. The coral SML microbiome in inner reefs in Bermuda is more exposed to natural temperature fluctuations (72, 75, 76). Therefore, a fluctuating profile is crucial to accurately describe the microbiome from that reef zone using temperature as an extrinsic factor. The coral SML microbiome of inner reefs is best predicted by the model scenarios that include high (SN-ST; GN-ST) or average (SN-GT; GN-GT) temperature fluctuations, since there is a significant loss in model fit when the temperature is kept constant (SN-CT; GN-CT), regardless of the associated network. In contrast, the SML microbiome of corals in the outer reefs were modeled using any temperature or network profile but the model outputs were less accurate when a warmer and more fluctuating temperature profile was applied.

The model shows that the coral SML microbiome from a fluctuating environment is more sensitive to the temperature profile used to achieve accurate predictions of the microbial relative abundances. This indicates that temperature is exerting a stronger and more directional pressure on the microbiome of inner reef corals, as they experience more pronounced temperature fluctuations than corals from the outer reefs (annual temperature range of 13 to 15°C in inner patch reefs and 10°C in outer reefs) (72). The sample data show that the coral SML microbiome structure is more homogenous across host individuals of *P. strigosa* in inner reefs than in outer reefs. Stability in the microbiome among colonies exposed to environmental stress is characteristic of a directional, rather than stochastic, response to pressure (77). Microbiome stability under environmental fluctuations was an unexpected finding. Previously, the microbiome of *P. strigosa* showed high variability under stress conditions *in situ*, compared to *Diploria labyrinthiformis*, a closely related species (78). However, the microbiome of *P. strigosa* shows low intercolony variation within the same site over time in the Caribbean (79), indicating that the microbiome associated with different colonies of *P. strigosa* acclimates similarly to temporal variability, which could explain the stability among inner reef corals. The local temperature profile may also influence the differences in the relative abundances between the microbiomes from inner and outer reefs. *Chlamydiia* and *Flavobacteriia* are driving the difference in the microbiomes between the two reef zones. The abundance of *Flavobacteriia* increased in the microbiome of *P. strigosa* in the summer compared to winter (78) and both taxa have been associated with elevated temperatures in coral reef environments (27, 33, 80–82). The overrepresentation of members of these classes in the microbiomes of corals from inner reefs of Bermuda may be related to the warmer local temperature profile but can also be simultaneously influenced by other factors (e.g., fluctuations in dissolved organic carbon [DOC]) (83, 84). The coral holobiont responds to fluctuations in abiotic factors such as light availability, pCO_2_, total alkalinity, pH, dissolved inorganic carbon (DIC) (76), and DOC (12, 14, 85). These could also be driving the community structure in the SML microbiome of corals and be potential extrinsic factors to be added to our model. However, in Bermuda, many of these variables are correlated with seasonal temperature fluctuations (76), which could partially explain why seawater temperature as the only abiotic factor is sufficient to accurately model the SML microbiome of corals.

The specificities of the microbial networks associated with each reef zone are also in accordance with trends shown in the model and the microbial community composition. Cooccurrence networks are able to detect small-scale environmental differences and show network specificity to each environment (86). The coral SML microbiome from outer reefs was more variable between individual corals, but the microbial network was highly structured, because many microbial classes had high eigen centrality, whereas betweenness centrality across taxa was low. High eigen centrality and low betweenness centrality are characteristic of keystone taxa in microbial networks (86). The milder environment in outer reefs could be releasing the coral SML microbiome from constant microbial community turnover caused by external disturbances, allowing the microbial community to establish several different cooccurrence patterns and generating more hubs of keystone taxa in the network, e.g., in the microbial network of outer reefs eight classes had an eigen centrality of >0.75 compared to three classes in the network of inner reefs. A host-associated microbial network that is less disturbed is characterized by nodes that are more interdependent and is more vulnerable to targeted disturbances, since the removal of hub species caused a greater disruption of the network diameter (87). The microbial network associated with the SML microbiome of inner reef corals showed the opposite structure, composed of high values of betweenness centrality and low eigen centrality. Host-associated microbial networks characterized by high betweenness centrality and low occurrence of large hubs of interconnected microbial taxa are considered resilient because the removal of nodes would not greatly impact the connectivity of the others (88). Therefore, the coral SML microbiome in the inner reef zone in Bermuda is organized in a network structure that potentially confers resilience to the microbial community exposed to environmental disturbances, while the outer reefs provide a more stable environment that is conducive to a tightly connected microbial network.

Coral reefs provide a variety of habitats characterized by different environmental conditions, which affect the biological community from the scale of macro- to microorganisms (4, 12, 89, 90). Our results are in agreement with studies that show that the reef zone in which the coral colony resides is a major factor shaping the coral microbiome composition (46, 48), particularly across areas exposed to different thermal regimes (47). The spatial gradient in temperature profiles across the reef zones in Bermuda is coupled with documented variations in coral growth, calcification (76), and reproductive processes (75, 91, 92). We showed that the coral SML microbiome responds to the local thermal environment in the coral reefs of Bermuda.

### Model applicability and future directions.

Coral microbiome dysbiosis (i.e., shifts in the community structure or a complete loss of microbial symbionts) caused by changes in the environment is a key mechanism in the decrease of coral health worldwide (13, 29, 50, 93). The lack of data sets describing long-term coral-microbe dynamics is interfering with successful predictions of how environmental change will affect the coral holobiont (43). Metagenomics followed by modeling and prediction are highlighted as main analytical tools to disentangle coral disease causation and to identify the successful application of mitigation strategies (94). The dynamic model we developed and validated using sampled metagenomes has the potential to be applied in microbial ecology research and coral reef management. A caveat to this study is that only one time point was used to validate the dynamic model. A time series of the coral microbiome *in situ* will further improve the model with regard to the temporal fluctuations in the microbiome structure. However, the model produced accurate outcomes across multiple scenarios of temperature and network profiles, suggesting that the model is very robust. We modeled the coral SML microbiome at the class level to obtain a number of taxa that are large enough to be representative of both two reef zones and achieve model accuracy, since all of the differential equations for each taxon are solved simultaneously. After the rare taxa (relative abundance < 1%) were removed, we had more than 70% of the total richness identified in the metagenomes represented in our model. We note that our model can be implemented using other taxonomic levels (e.g., order, family, and genera), if the parameters that are necessary to calculate growth rates *R*(*T*) are known for each taxon. We recommend the use of a cutoff value to remove the rare taxa and maintain the accuracy of the predicted relative abundances.

The model is relatively simple to use and to interpret and can be used to simulate the changes in the SML microbiome in response to seasonal temperature fluctuations (Fig. 7). Coral species exhibit a wide variation in thermal resilience (95–97) and mucus production and composition (98), which indirectly shapes the associated SML microbiome. The coral *Porites astreoides*, for example, goes through cycles of mucus aging and shedding that affects the microbiome dynamics (99). Therefore, the model developed here could be used to identify whether the microbiomes of other species are affected by temperature like the *P. strigosa* SML microbiome. We recommend at least one annual collection of the mucus microbiome for metagenomic analysis across different reef environments for model calibration. This approach will allow for a level of resolution specific to different areas across the reef that might require distinct management decisions. For example, the model can be used to evaluate whether different environments across the reef are more susceptible to disease or dysbiosis due to a predicted change in the relative abundances of microbial taxa under specific temperature conditions. Our model can also be used to describe the microbiome associated with the coral tissue and skeleton following the same workflow that we developed for the SML microbiome (Fig. 7). However, since each compartment provides a specific microhabitat to the microbial community (44, 45), the coral tissue and skeleton microbiomes may respond differently to temperature fluctuations compared to the SML microbiome (44). We encourage the application of this model to other compartments of the coral microbiome (i.e., tissue and skeleton), as well as different coral species and coral reef systems, to compare whether temperature remains the primary ecological driver of the host-associated microbiome compared to the microbial network.

## MATERIALS AND METHODS

### *In situ* collections.

The mucus from *P. strigosa* was collected from six colonies from the inner and outer reef zones (*n* = 12 colonies total) in May and June 2017. Each reef zone was replicated across three reef sites (Fig. 1). The SML microbiome of *P. strigosa* colonies (diameter, 10 to 15 cm) was collected using a “supersucker,” a two-way 50-ml syringe filled with 0.02-μm-filtered seawater (100). The filtered seawater is flushed across the coral surface, dislodging the mucus and associated microbes, which are then sucked up via the recirculating tube, and the resulting sample pushed through a 0.22-μm Sterivex (EMD Millipore) for DNA extraction. We collected 200 ml of coral mucus diluted in sterile seawater (four supersuckers) per colony to increase DNA concentration per sample. The reef water microbiome was also analyzed to control for contamination in the mucus samples (results not shown). The collections were performed via SCUBA diving at a depth of 4 to 6 m.

### Metagenomics analysis.

Microbial DNA from the coral mucus was extracted using a modified Macherey-Nagel protocol from 0.22-μm Sterivex using NucleoSpin column for purification. DNA was stored at –20°C until quantification with Qubit (Thermo Fisher Scientific) (14). The Swift kit 2S plus (Swift Biosciences) was used for library preparation since it provides good results from small amounts of input DNA, characteristic of microbial samples collected from the surface of the host (100–102). All samples were sequenced by the Dinsdale lab on Illumina MiSeq at San Diego State University (103). The Illumina MiSeq is one of the best sequencing technologies for short genomes, such as those associated with bacteria and archaea, and provides longer reads compared to the Illumina HiSeq (104, 105). We described the proportional abundance of *Bacteria* and *Archaea* in the coral mucus microbiome using shotgun metagenomics (62, 64). The sequenced DNA was analyzed for quality control using PrinSeq (106) before annotation. The forward and reverse reads were paired using PEAR (107). The sequencing depth ranged from 582,582 to 1,256,934 reads per metagenome (see Table S4 in the supplemental material). FOCUS (108), which is a K-mer-based approach, was used to annotate taxa. FOCUS has been identified as one of the top profiling analysis tools by CAMI (109). The number of sequence hits for each microbial taxon is represented as the relative abundance by calculating the proportion of sequence hits for that class over the total number of sequences annotated for that metagenome.

10.1128/mBio.02691-19.6 TABLE S4 Results of PEAR analysis on the metagenomes from the coral mucus collected from inner and outer reefs (*n* [total] = 12). Both forward and reverse reads files were used. Download Table S4, DOCX file, 0.01 MB. Copyright © 2020 Lima et al. 2020 Lima et al. This content is distributed under the terms of the Creative Commons Attribution 4.0 International license.

### Statistical analysis of the sample data.

Statistical analyses were conducted using PRIMER v7 plus PERMANOVA and R (R Project for Statistical Computing). Significant differences in the relative abundances of classes in the coral microbial communities sampled from inner and outer reefs were identified by permutational multivariate analysis of variance (PERMANOVA) using Bray-Curtis distances of normalized relative abundance obtained using a fourth-root transformation. A principal coordinate analysis was created to visualize the separation of the coral microbiome between inner and outer reefs and the most important taxa driving the cluster by plotting the vectors corresponding to Spearman’s correlation indices. A SIMPER analysis was performed to identify the taxa responsible for the similarity of the microbiomes within reef zones and dissimilarity between reef zones. The microbial network was constructed for each metagenomic data set the taxonomic pairwise Spearman correlation matrix calculated in R. The matrix was calculated for each reef zone, and the network correlation coefficients were used in the dynamic model described in under “Mathematical model” below. The Python packages pandas (110) and networkx (111) were used to test for subclustering of the networks and identified that each network remains a single connected component. The R package igraph (112) was used to construct a network using the microbial taxa at class level as nodes and the Spearman correlation values as edge weights. The calculated diameter of the network was unweighted. Taxonomic cooccurrences that met or exceeded the preset correlation threshold were kept, while all other values were transformed to 0. The psych package (113) was used to calculate the *P* value for all pairwise coefficients. All pairwise coefficients from which the *P* value exceeded 0.001 were discarded from the analysis. To identify taxa that occupy important structures of the microbial network, the R package igraph was used to calculate the eigenvector and betweenness centrality. Eigen centrality identifies highly connected nodes that are connected to other highly connected nodes (114, 115). Betweenness centrality calculates the shortest path through a network and keeps record of how many times a node in a network is traversed (74). If a node is traversed frequently, the node in the network is considered to sit at a position that is important in facilitating the connectivity of the network. If the taxon has a high betweenness centrality, then it sits at a position in the network that is responsible for facilitating correlations between different taxa. Without the presence of that taxon, the network loses the architecture that binds it together in an ordered way.

### Mathematical model.

In an isolated environment, we assume that each microbial class, $B_{i}$, grows according to the classical logistic growth equations; however, growth rate, *R_i_*(*T*(*t*)), is represented as a function of the environmental temperature, *T*(*t*), which changes over time. Since each microorganism has a distinct range of ideal temperatures for its growth, we consider the growth rate, *R_i_*(*T*(*t*)), to be normally distributed with mean at the midpoint of the range of the ideal temperature as follows.

$$R(T)=R_{\text{max}}\cdot \frac{1}{\sqrt{2\pi \sigma^{2}}}\cdot e^{-\frac{{\left(T-\mu \right)}^{2}}{2\sigma^{2}}}$$

Here, *R*_max_ is the maximum growth rate, μ is the mean ideal growth temperature, and σ is the range of ideal temperatures. These values are specific to each microbial taxon and are summarized in the Table S5 in the supplemental material. The values used in the calculation of maximum growth rates were obtained from the literature for cultured representatives of each microbial class.

10.1128/mBio.02691-19.7 TABLE S5 Growth rate values by microbial class from cultured data obtained from the literature. Download Table S5, DOCX file, 0.01 MB. Copyright © 2020 Lima et al. 2020 Lima et al. This content is distributed under the terms of the Creative Commons Attribution 4.0 International license.

Variation in temperature is captured using a sinusoidal function (59),

T(t)=M+A⋅sin⁡(ω⋅t+θ),

where *M* and *A* represent the mean and the amplitude of the temperature profile. ω is related to the period of the periodic temperature profile, i.e., the period is 2π/ω and θ represents the phase shift of the temperature used to make the temperature equation more accurately reflect the temperature conditions in the reefs of Bermuda according to the literature (72, 75, 76). The differences in reef zone environment are represented with different values for *M* and *A*, as described under “Network and temperature profiles” below.

When the microorganisms reside in a community together, such as in the coral mucus, there are two major potential effects each microbial taxon faces due to the presence of one another: (i) all microbes compete for the common resources denoted by the total carrying capacity (*k*), and (ii) interactions among microbes in a network alter the net growth rate of each other. To introduce the first effect into the model, we assumed that all taxa compete identically for the common resources since their relative competition coefficients are not well established. The second effect is introduced by altering the net growth rate of each microbial taxon with all other components of the microbiome according to its network correlation coefficients. The model we use is as follows:

$$\frac{dB_{i}}{\mathrm{dt}}=R_{i}\left(T\left(t\right)\right)\cdot B_{i}\cdot \left(1-\frac{\sum {}_{j=1}^{n}B_{j}}{k}\right)\cdot \left(1+\overset{n}{\underset{j=1}{\sum }}\eta_{\mathrm{ij}}\cdot B_{j}\right),i=1,2, . . .N.$$

Here, *N* is the total number of taxa, *k* represents the carrying capacity, and *η_ij_* represents the network correlation coefficient between the *i*th class and the *j*th class of bacteria.

### Network and temperature profiles.

The model uses network correlation coefficients [*η_ij_* and temperature fluctuations over time *T*(*t*)] to predict the structure of the microbial community associated with coral mucus. Therefore, different profiles of microbial network and seawater temperature were selected in an ecological context of the coral reef system. For both network and temperature, we explored the specificities of each reef zone by including a “specific” profile. In addition, we also considered a “generalized” profile that represents the coral reef system on a broader scale, instead of according to the local zonation patterns. “Specific” and “generalized” profiles are used to evaluate whether the inclusion of values that represent the microbiome (network) and the environment (temperature) at a fine spatial scale are necessary to achieve accuracy in the model outputs.

The specific network profiles (SN) include the correlation coefficients (*η_ij_*) that represent the sample data collected in the specific reef zone being modeled. For example, to model the microbiome associated with inner reefs, the correlation coefficient (*η_ij_*) produced from all of the six metagenomes collected in inner reefs is used in the SN profile. In contrast, the generalized network profile (GN) uses the correlation coefficient (*η_ij_*) produced by all the metagenomes collected from both reef zones (*n* = 12).

The specific temperature profiles (ST) are produced by using yearly mean (M) and amplitude (A) in degree Celsius that are representative of each reef zone to calculate the temperature fluctuations as a function of time *T*(*t*). In the ST profile for the inner reef, the temperature mean and the amplitude are higher (M = 24, A = 7) than in the ST profile for the outer reef (M = 18, A = 5). Therefore, the ST profiles are developed to represent the local temperature regimes in terms of annual temperature fluctuations, in which the inner reef zone is a warmer and more fluctuating environment and the outer reef zone is a milder and more stable thermal environment. The generalized temperature profile (GT) used the average between the parameters in the ST profiles (M = 21, A = 6). In all of the temperature profiles, we used ω = 2π/365 to account for the annual variability of temperature according to seasons (Fig. S2). Constant temperature profiles (CT) are also considered to evaluate the effect of temperature fluctuations on the model outputs. The CT profiles use the mean temperatures specific to each reef zone, (inner: M = 24, A = 0; outer: M = 18, A = 0).

10.1128/mBio.02691-19.2 FIG S2 Fluctuating temperature profiles used in the model scenarios. The specific temperature profiles (ST) are produced by using yearly mean (M) and amplitude (A) that are representative of each reef zone to calculate the temperature fluctuations as a function of time, *T*(*t*). In the ST profile for the inner reef, the temperature mean and the amplitude are higher (M = 24, A = 7) than in the ST profile for the outer reef (M = 18, A = 5). The generalized temperature profile (GT) used the average between the parameters in the ST profiles (M = 21, A = 6). In all of the temperature profiles, we used *t* = 365 days to account for the annual variability of temperature according to seasons. Download FIG S2, TIF file, 2.5 MB. Copyright © 2020 Lima et al. 2020 Lima et al. This content is distributed under the terms of the Creative Commons Attribution 4.0 International license.

### Model application to identify drivers of microbiome dynamics.

The mathematical model we developed considers both intrinsic and extrinsic factors affecting the coral mucus microbiome. The intrinsic factor is the microbial interaction within the microbiome, characterized by the network analysis, and the extrinsic factor is environmental temperature. To determine the key drivers governing coral mucus microbiome composition across reef zones, we evaluate six different model scenarios (i.e., different combination of network and temperature profiles):

(i) Specific network, specific temperature (SN-ST): both the temperature profile and network parameters used are specific to each reef zone.

(ii) Specific network, generalized temperature (SN-GT): the network profile is specific for each reef zone, but the temperature profile is generalized.

(iii) Specific network, constant temperature (SN-CT): the network profile is specific to each reef zone, but temperature remains constant at the mean specified for each reef zone.

(iv) Generalized network, specific temperature (GN-ST): the network profile is generalized, but the temperature profile is specific for each reef zone.

(v) Generalized network, generalized temperature (GN-GT): the network profile and the temperature profile are generalized.

(vi) Generalized network, constant temperature (GN-CT): the network profile is generalized, and the temperature remains constant at the mean specified for each reef zone.

### Statistical analysis of the model output data.

The microbial relative abundances generated from the model were compared to the sample data from inner and outer reefs using a linear regression (in R Project for Statistical Computing). A Wald linear hypothesis test was performed on the parameters generated by the linear regression analysis (i.e., slope and intercept). If the model is an accurate predictor of the coral microbiome, the slope will not be statistically different from 1, and the intercept will not be statistically different from 0. Each model scenario was tested by the fourth-root transformation of sample data, and model relative abundances were applied to achieve normality (Shapiro-Wilk test); a linear regression analysis was then performed and tested. Model components associated with changes in accuracy (*R*^2^) of the model outputs are considered key factors shaping the coral microbiome structure. For example, if all model scenarios that include “ST” are more accurate than the others, regardless of the network profile used, then the local temperature profile is a key factor. Therefore, temperature is a primary driver, and microbial interactions is a secondary driver shaping the coral microbiome structure. If there are no differences in accuracies across model scenarios, then it is assumed that all factors have the same impact on the microbial community. Therefore, by comparing the model outputs generated by different combinations of network and temperature profiles, the model was applied to investigate the drivers of the coral-microbial community dynamics.

### Data availability.

The metagenomic data from this study is publicly available in the SRA database as BioProject PRJNA595374 (https://www.ncbi.nlm.nih.gov/bioproject/595374) and in MG-RAST as public study SDSU_BIOS_2017 (mgp81589; https://www.mg-rast.org/linkin.cgi?project=mgp81589). The scripts used for the statistical analysis in R, Python, and PRIMER are publicly available as a GitHub repository under “MichealBReed/Microbiome_model” (https://github.com/MichealBReed/Microbiome_model).
