# Supplementary material for: Modeling of the Coral Microbiome: the Influence of Temperature and Microbial Network
Source: mBio. 2020 Mar 3;11(2):e02691-19. doi: 10.1128/mBio.02691-19 (PMC7064765; doi:10.1128/mBio.02691-19)
Supplement: TABLE S1 [file mBio.02691-19-st001.docx]

Table S1. Permutational MANOVA (PERMANOVA) table of results using a Bray Curtis similarity matrix of fourth-root transformed relative abundances from metagenomes collected from outer and inner reefs. The permutation method selected was unrestricted permutation of raw data using 999 permutations. Reef zone was treated as a fixed factor of two levels.

| Source | df | SS | MS | Pseudo-F | P(perm) | Unique perms |
| --- | --- | --- | --- | --- | --- | --- |
| Reef | 1 | 642.35 | 642.35 | 7.7879 | 0.004 | 404 |
| Residuals | 10 | 824.81 | 82.481 |  |  |  |
| Total | 11 | 1467.2 |  |  |  |  |
