# Supplementary material for: Modeling of the Coral Microbiome: the Influence of Temperature and Microbial Network
Source: mBio. 2020 Mar 3;11(2):e02691-19. doi: 10.1128/mBio.02691-19 (PMC7064765; doi:10.1128/mBio.02691-19)
Supplement: TABLE S2 [file mBio.02691-19-st002.docx]

Table S2. Similarity Percentages (SIMPER) of species contributions using a Bray Curtis similarity matrix of fourth-root transformed relative abundances from metagenomes collected from outer and inner reefs. Each reef zone was treated as a factor group. Cut off for low contributions: 90.00 %. The SIMPER results of similarity within groups is shown for outer reef (S2a), inner reefs (S2b) and a comparison between the two reef zones (S2c).

Table S2a. Group Outer, average similarity: 84.70.

| Microbial taxa | Average Abundance | Average Similiarity | Similarity/SD | Percentual Contribution | Cumulative Percentual Contribution |
| --- | --- | --- | --- | --- | --- |
| Alphaproteobacteria | 2.37 | 8.95 | 10.32 | 10.57 | 10.57 |
| Bacilli | 1.94 | 7.55 | 36.40 | 8.91 | 19.48 |
| Gammaproteobacteria | 1.71 | 6.42 | 26.39 | 7.58 | 27.06 |
| Thaumarchaeota | 1.60 | 6.23 | 27.32 | 7.36 | 34.42 |
| Clostridia | 1.56 | 5.91 | 21.62 | 6.98 | 41.40 |
| Spirochaetia | 1.52 | 5.76 | 34.84 | 6.80 | 48.20 |
| Actinobacteria | 1.49 | 5.69 | 13.29 | 6.72 | 54.92 |
| Betaproteobacteria | 1.37 | 5.30 | 20.58 | 6.26 | 61.18 |
| Thermoprotei | 1.27 | 4.58 | 14.39 | 5.41 | 66.60 |
| Mollicutes | 1.20 | 4.35 | 13.40 | 5.13 | 71.73 |
| Methanobacteria | 1.12 | 3.96 | 7.99 | 4.68 | 76.41 |
| Halobacteria | 0.97 | 3.57 | 18.47 | 4.21 | 80.62 |
| Deltaproteobacteria | 0.86 | 2.71 | 3.96 | 3.21 | 83.82 |
| Methanomicrobia | 0.77 | 2.55 | 3.60 | 3.01 | 86.83 |
| Cyanobacteria | 1.04 | 2.47 | 0.79 | 2.91 | 89.74 |
| Epsilonproteobacteria | 0.70 | 2.41 | 8.48 | 2.84 | 92.58 |

Table S2b. Group Inner, average similarity: 92.89.

| Microbial taxa | Average Abundance | Average Similarity | Similarity/SD | Percentual Contribution | Cumulative Percentual Contribution |
| --- | --- | --- | --- | --- | --- |
| Alphaproteobacteria | 2.14 | 7.10 | 67.20 | 7.64 | 7.64 |
| Bacilli | 1.75 | 5.73 | 43.19 | 6.17 | 13.81 |
| Gammaproteobacteria | 1.67 | 5.39 | 16.85 | 5.80 | 19.61 |
| Mollicutes | 1.63 | 5.35 | 26.43 | 5.76 | 25.37 |
| Spirochaetia | 1.56 | 5.18 | 62.13 | 5.58 | 30.96 |
| Clostridia | 1.53 | 5.03 | 29.40 | 5.42 | 36.37 |
| Thaumarchaeota | 1.52 | 5.02 | 45.16 | 5.41 | 41.78 |
| Chlamydiia | 1.51 | 4.92 | 30.82 | 5.30 | 47.08 |
| Thermoprotei | 1.39 | 4.58 | 36.85 | 4.94 | 52.01 |
| Actinobacteria | 1.41 | 4.57 | 20.82 | 4.92 | 56.93 |
| Betaproteobacteria | 1.39 | 4.46 | 20.66 | 4.80 | 61.74 |
| Flavobacteriia | 1.36 | 4.44 | 42.40 | 4.78 | 66.52 |
| Methanobacteria | 1.34 | 4.39 | 32.17 | 4.73 | 71.25 |
| Planctomycetia | 1.33 | 4.35 | 35.72 | 4.68 | 75.93 |
| Cyanobacteria | 1.31 | 4.07 | 10.58 | 4.38 | 80.31 |
| Halobacteria | 1.20 | 3.90 | 17.84 | 4.20 | 84.51 |
| Deinococci | 1.09 | 3.51 | 29.42 | 3.78 | 88.28 |
| Deltaproteobacteria | 1.06 | 3.29 | 11.96 | 3.54 | 91.82 |

Table S2c. Groups Outer and Inner, average dissimilarity = 18.16

| Microbial taxa | Outer Av. Abundance | Inner Av. Abundance | Average Dissimilarity | Dissimilarity/SD | Percentual Contribution | Cumulative Percentual Contribution |
| --- | --- | --- | --- | --- | --- | --- |
| Chlamydiia | 0.52 | 1.51 | 1.80 | 1.79 | 9.92 | 9.92 |
| Deinococci | 0.13 | 1.09 | 1.74 | 3.11 | 9.61 | 19.53 |
| Flavobacteriia | 0.61 | 1.36 | 1.37 | 1.65 | 7.53 | 27.06 |
| Sphingobacteriia | 0.71 | 0.00 | 1.30 | 1.39 | 7.15 | 34.21 |
| Planctomycetia | 0.62 | 1.33 | 1.30 | 1.37 | 7.14 | 41.35 |
| Cyanobacteria | 1.04 | 1.31 | 1.08 | 1.16 | 5.95 | 47.30 |
| Thermotogae | 0.66 | 0.91 | 0.99 | 1.16 | 5.45 | 52.75 |
| Cytophagia | 0.17 | 0.55 | 0.99 | 1.30 | 5.43 | 58.19 |
| Bacteria | 0.00 | 0.46 | 0.83 | 1.25 | 4.58 | 62.77 |
| Mollicutes | 1.20 | 1.63 | 0.78 | 2.56 | 4.30 | 67.07 |
| Bacteroidia | 0.32 | 0.00 | 0.60 | 0.70 | 3.31 | 70.38 |
| Methanomicrobia | 0.77 | 0.84 | 0.60 | 1.20 | 3.30 | 73.68 |
| Deltaproteobacteria | 0.86 | 1.06 | 0.51 | 1.46 | 2.80 | 76.48 |
| Alphaproteobacteria | 2.37 | 2.14 | 0.46 | 1.77 | 2.52 | 79.00 |
| Halobacteria | 0.97 | 1.20 | 0.43 | 2.18 | 2.35 | 81.35 |
| Methanobacteria | 1.12 | 1.34 | 0.41 | 1.29 | 2.24 | 83.59 |
| Epsilonproteobacteria | 0.70 | 0.58 | 0.37 | 0.81 | 2.06 | 85.65 |
| Bacilli | 1.94 | 1.75 | 0.36 | 2.70 | 1.97 | 87.62 |
| Thermoprotei | 1.27 | 1.39 | 0.36 | 2.30 | 1.96 | 89.57 |
| Chlorobia | 0.14 | 0.08 | 0.34 | 0.61 | 1.89 | 91.47 |
