# Supplementary figures and images for: Modeling of the Coral Microbiome: the Influence of Temperature and Microbial Network
Source: mBio. 2020 Mar 3;11(2):e02691-19. doi: 10.1128/mBio.02691-19 (PMC7064765; doi:10.1128/mBio.02691-19)

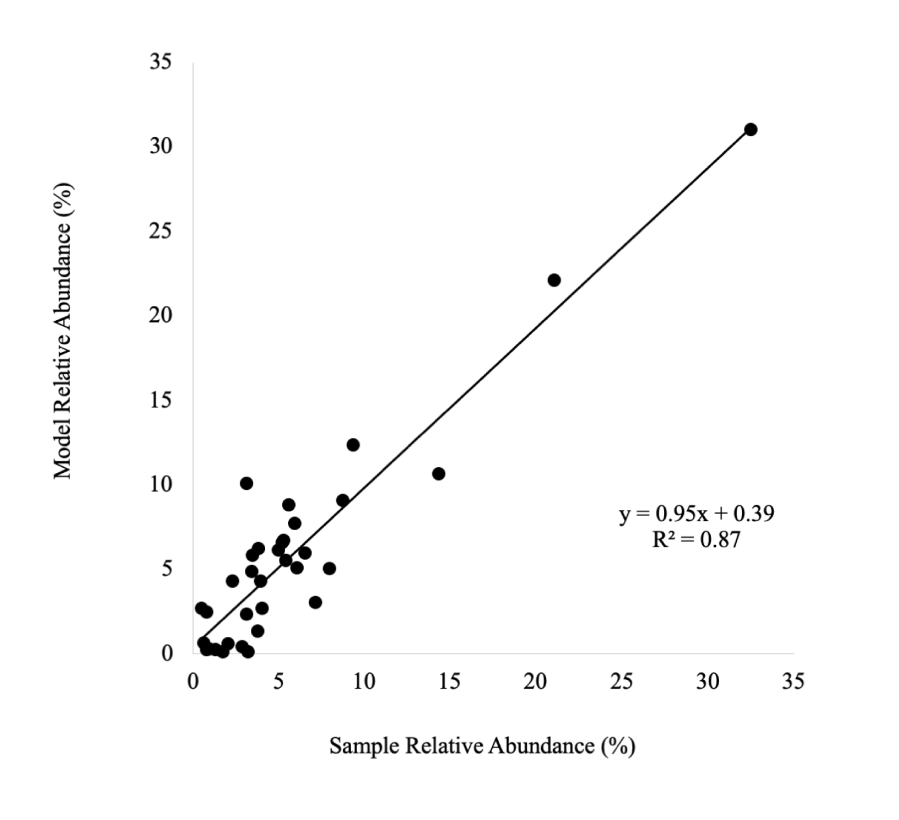

Supplement: FIG S1 [file mBio.02691-19-sf001.tif]

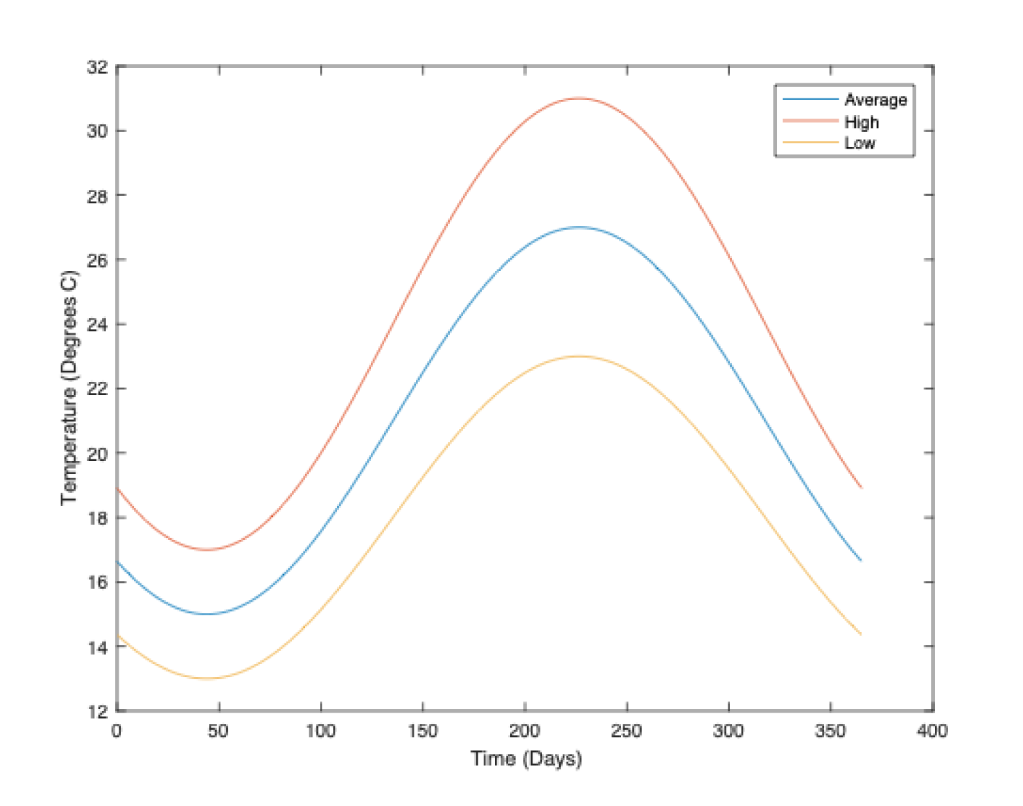

Supplement: FIG S2 [file mBio.02691-19-sf002.tif]
