# Supplementary material for: Modeling of the Coral Microbiome: the Influence of Temperature and Microbial Network
Source: mBio. 2020 Mar 3;11(2):e02691-19. doi: 10.1128/mBio.02691-19 (PMC7064765; doi:10.1128/mBio.02691-19)
Supplement: TABLE S3 [file mBio.02691-19-st003.docx]

Table S3. Results of the linear regression analysis between the model outputs and observed data after fourth-root transformation to achieve normality.

|  | R - squared | | Coefficients (Intercept, Model ~ Data) | | | | ANOVA table (df = 1,15) | | | |
| --- | --- | --- | --- | --- | --- | --- | --- | --- | --- | --- |
| Model case | Multiple | Adjusted | Estimate | Std. Error | t value | Pr(>\|t\|) | Mean Sq | F value | Pr(>F) |  |
| Inner SN-ST | 0.57 | 0.54 | -0.55, 1.32 | 0.45, 0.30 | -1.21, 4.41 | 0.24, 0.0005 | 1.63 | 19.46 | 0.0005 |  |
| Inner SN-GT | 0.64 | 0.62 | -0.83, 1.50 | 0.43, 0.29 | -1.9, 5.16 | 0.08,  0.0001 | 2.07 | 26.62 | 0.0001 |  |
| Inner SN-CT | 0.14 | 0.08 | 0.27, 0.73 | 0.71, 0.47 | 0.38, 1.55 | 0.71, 0.14 | 0.50 | 2.41 | 0.1414 |  |
| Inner GN-ST | 0.51 | 0.48 | -0.45, 1.25 | 0.47, 0.31 | -0.92, 3.98 | 0.37, 0.001 | 1.45 | 15.86 | 0.001 |  |
| Inner GN-GT | 0.58 | 0.55 | -0.70, 1.42 | 0.47, 0.31 | -1.5, 4.56 | 0.15, 0.0004 | 1.86 | 20.74 | 0.0004 |  |
| Inner GN-CT | 0.16 | 0.11 | 0.20, 0.78 | 0.69, 0.46 | 0.29, 1.69 | 0.78, 0.11 | 0.57 | 2.87 | 0.11 |  |
| Outer SN-ST | 0.69 | 0.67 | 0.02, 0.96 | 0.23, 0.16 | 0.10, 5.77 | 0.921, 0.0001 | 2.45 | 33.33 | 0.0001 |  |
| Outer SN-GT | 0.5912 | 0.56 | 0.25, 0.84 | 0.26, 0.18 | 0.96, 4.66 | 0.35, 0.0003 | 1.88 | 21.69 | 0.0003 |  |
| Outer SN-CT | 0.69 | 0.67 | -0.19, 1.06 | 0.26, 0.18 | -0.72, 5.79 | 0.48, 0.0001 | 3.01 | 33.57 | 0.0001 |  |
| Outer GN-ST | 0.69 | 0.67 | 0.023,  0.96 | 0.24, 0.16 | 0.10, 5.8 | 0.92, 0.0001 | 2.47 | 33.71 | 0.0001 |  |
| Outer GN-GT | 0.59 | 0.56 | 0.24,  0.84 | 0.26, 0.18 | 0.94, 4.64 | 0.36, 0.0001 | 1.88 | 21.49 | 0.0001 |  |
| Outer GN-CT | 0.70 | 0.68 | -0.19, 1.07 | 0.26, 0.18 | -0.75, 5.88 | 0.467, 0.0001 | 3.05 | 34.54 | 0.0001 |  |
