# Supplementary material for: Modeling of the Coral Microbiome: the Influence of Temperature and Microbial Network
Source: mBio. 2020 Mar 3;11(2):e02691-19. doi: 10.1128/mBio.02691-19 (PMC7064765; doi:10.1128/mBio.02691-19)
Supplement: TABLE S4 [file mBio.02691-19-st004.docx]

Table S4. Results of PEAR analysis on the metagenomes from the coral mucus collected from inner and outer reefs (n total = 12). Both forward and reverse reads files were used.

| Sample ID | Assembled reads | Total reads | Percent assembled |
| --- | --- | --- | --- |
| Inner_1 | 827,765 | 837,164 | 98.8 |
| Inner_2 | 1,152,297 | 1,160,262 | 99.3 |
| Inner_3 | 737,941 | 747,204 | 98.7 |
| Inner_4 | 787,513 | 792,229 | 99.4 |
| Inner_5 | 919,584 | 929,838 | 98.9 |
| Inner_6 | 972,345 | 977,873 | 99.4 |
| Outer_1 | 823,807 | 838,796 | 98.2 |
| Outer_2 | 732,345 | 746,773 | 98.1 |
| Outer_3 | 574,195 | 582,582 | 98.5 |
| Outer_4 | 608,263 | 615,214 | 98.8 |
| Outer_5 | 725,498 | 734,758 | 98.7 |
| Outer_6 | 1,251,217 | 1,256,934 | 99.5 |
