# Supplementary material for: Modeling of the Coral Microbiome: the Influence of Temperature and Microbial Network
Source: mBio. 2020 Mar 3;11(2):e02691-19. doi: 10.1128/mBio.02691-19 (PMC7064765; doi:10.1128/mBio.02691-19)
Supplement: TABLE S5 [file mBio.02691-19-st005.docx]

Table S5. Growth rate values by microbial class from cultured data obtained from the literature.

| Class | $R_{max}$ | $\sigma$ | $\mu$ | Reference |
| --- | --- | --- | --- | --- |
| Actinobacteria | 0.5 | 5 | 22 | Brown, J. Howard. "Bergey's manual of determinative bacteriology." (1939): 404-405. |
| Alphaproteobacteria | 1.1 | 8 | 26 | Ferrera, Isabel, et al. "Comparison of growth rates of aerobic anoxygenic phototrophic bacteria and other bacterioplankton groups in coastal Mediterranean waters." *Appl. Environ. Microbiol.* 77.21 (2011): 7451-7458. |
| Bacilli | 1.6 | 20 | 34 | Warth, Alan D. "Relationship between the heat resistance of spores and the optimum and maximum growth temperatures of Bacillus species." *Journal of Bacteriology* 134.3 (1978): 699-705. |
| Betaproteobacteria | 0.9 | 5 | 27 | Teira, Eva, et al. "Betaproteobacteria growth and nitrification rates during long-term natural dissolved organic matter decomposition experiments." *Aquatic Microbial Ecology* 63.1 (2011): 19-27. |
| Chlamydiia | 1 | 5 | 30 | Brown, J. Howard. "Bergey's manual of determinative bacteriology." (1939): 404-405. |
| Clostridia | 0.75 | 1 | 33 | Sottile, William, and R. J. Zabransky. "Comparative growth rates of selected anaerobic species in four commonly used broth media." *Antimicrobial agents and chemotherapy* 11.3 (1977): 482-490. |
| Cyanobacteria | 1.75 | 10 | 35 | Lürling, Miquel, et al. "Comparison of cyanobacterial and green algal growth rates at different temperatures." *Freshwater Biology* 58.3 (2013): 552-559. |
| Deltaproteobacteria | 0.1 | 5 | 25 | Badziong, Werner, and Rudolf K. Thauer. "Growth yields and growth rates of Desulfovibrio vulgaris (Marburg) growing on hydrogen plus sulfate and hydrogen plus thiosulfate as the sole energy sources." *Archives of microbiology* 117.2 (1978): 209-214. |
| Flavobacteriia | 0.5 | 5 | 22 | Brown, J. Howard. "Bergey's manual of determinative bacteriology." (1939): 404-405. |
| Gammaproteobacteria | 0.5 | 4 | 22 | Ferrera, Isabel, et al. "Comparison of growth rates of aerobic anoxygenic phototrophic bacteria and other bacterioplankton groups in coastal Mediterranean waters." *Appl. Environ. Microbiol.* 77.21 (2011): 7451-7458. |
| Halobacteria | 1 | 10 | 40 | Gonzalez, Orland, et al. "Systems analysis of bioenergetics and growth of the extreme halophile Halobacterium salinarum." *PLoS computational biology* 5.4 (2009): e1000332. |
| Methanobacteria | 1 | 2 | 34 | Zhao, Yizhang, et al. "Isolation and characterization of a fast-growing, thermophilic Methanobacterium species." *Appl. Environ. Microbiol.* 52.5 (1986): 1227-1229. |
| Mollicutes | 1 | 15 | 34 | Konai, Meghnad, et al. "Temperature ranges, growth optima, and growth rates of Spiroplasma (Spiroplasmataceae, class Mollicutes) species." *Current microbiology* 32.6 (1996): 314-319. |
| Planctomycetia | 0.7 | 8 | 27 | Lage, Olga Maria, and Joana Bondoso. "Bringing Planctomycetes into pure culture." *Frontiers in microbiology* 3 (2012): 405. |
| Spirochaetia | 0.75 | 5 | 25 | Brown, J. Howard. "Bergey's manual of determinative bacteriology." (1939): 404-405. |
| Thaumarchaeota | 0.8 | 5 | 25 | Stieglmeier, Michaela, Ricardo JE Alves, and Christa Schleper. "The phylum thaumarchaeota." *The Prokaryotes: other major lineages of bacteria and the archaea* (2014): 347-362. |
| Thermoprotei | 3 | 15 | 42 | Brown, J. Howard. "Bergey's manual of determinative bacteriology." (1939): 404-405. |
